# Effects of carbohydrate-electrolyte solutions with and without L-menthol on hydration and performance recovery following simulated firefighting exercise

**DOI:** 10.1080/15502783.2026.2676193

**Published:** 2026-05-19

**Authors:** Yi-Ju Hsu, Chin-Shan Ho, Chun-Hao Chang, Tsan-Wei Chuang, Shao-Hua Shang

**Affiliations:** a Graduate Institute of Sports Science, National Taiwan Sport University, Taoyuan City, Taiwan; b New Taipei City Fire Department, New Taipei City Government, New Taipei City, Taiwan

**Keywords:** Firefighting exercise, rehydration, electrolytes solution, menthol, fluid balance, performance recovery

## Abstract

**Background:**

Firefighting tasks performed in full protective equipment (PPE) impose extreme thermal and physical strain, providing a model of high-intensity exercise with substantial fluid and electrolyte loss. This study compared the effects of plain water (W), a carbohydrate-electrolyte solution (CES), and a menthol-supplemented carbohydrate-electrolyte solution (MCES) on hydration and performance recovery during the first hour of post-exercise recovery.

**Methods:**

Twenty-four male firefighters completed a standardized simulated firefighting protocol in protective clothing. Participants were randomly assigned to W, CES, or MCES (*n* = 8 each) and consumed 1.0 L of fluid during 60 minutes of recovery. Hydration status was evaluated using body-mass restoration, salivary osmolality, urine specific gravity, urine color, and urinary electrolytes. Renal markers, lactate clearance, cardiovascular and thermal responses, thirst ratings, and lower-limb explosive power were also assessed.

**Results:**

Body mass restoration showed significant group effects, with MCES greater than W and CES at 60 min (*p* < 0.05). Rehydration rate was higher for both CES and MCES than W (*p* < 0.05). Salivary osmolality and urinary sodium favored electrolyte solutions over water (*p* < 0.05), while urine specific gravity was lower in MCES compared with W and CES (*p* < 0.05). Blood urea nitrogen and BUN/creatinine ratios were higher in W than both electrolyte conditions at 30 and 60 min (*p* < 0.05). Lactate clearance was reduced in W compared with CES and MCES (*p* < 0.05). Jump height declined from pre- to post-recovery in the W and CES groups but was maintained within the MCES group.

**Conclusions:**

Carbohydrate-electrolyte solutions improved hydration compared with water. L-menthol did not enhance rehydration but maintained explosive performance, supporting its role as a perceptual adjunct to electrolyte strategies for recovery after exercise in protective clothing.

## 1. Introduction

Firefighting tasks performed in full personal protective equipment (PPE) represent an extreme model of high-intensity exercise under thermal strain. Firefighters are required to perform strenuous tasks in unfavourable conditions while wearing heavy and cumbersome PPE (~22 kg), along with additional gear (9–18 kg) [1,2]. In addition to the physical burden, they are exposed to significant heat stress from both environmental conditions and combustion products during rescue operations [3]. Although protective clothing provides thermal insulation, it also limits heat dissipation and elevates core and skin temperatures [4]. Heat stress is a common challenge in firefighting and contributes to fatigue and overexertion [5], which may impair neuromuscular performance, including strength and power output required for rescue tasks [6]. Therefore, simulated firefighting exercise provides a relevant model to examine the effects of thermal and metabolic stress on hydration and recovery.

High-intensity firefighting activity impairs thermoregulation through excessive sweating and fluid loss. Substantial amounts of water and electrolytes, including sodium, potassium, and chloride, are lost during such exertion [7–9]. Without adequate fluid replacement, thermoregulation becomes less efficient and the risk of dehydration-related fatigue increases [10]. In operational settings where continuous fluid intake is impractical, firefighters often consume plain water during brief recovery periods. While this may temporarily relieve thirst, excessive water intake without electrolyte replacement can disrupt fluid balance and impair recovery [11,12]. These challenges may reduce operational readiness and are comparable to those faced by athletes recovering from repeated high-intensity exercise.

Electrolyte-containing carbohydrate solutions are more effective than water for restoring fluid balance after exercise. They enhance fluid absorption, reduce diuresis, and support plasma volume restoration, thereby aiding metabolic and neuromuscular recovery [13]. In addition to hydration, thermal comfort plays an important role in post-incident recovery. Menthol, a non-thermal cooling agent, has been shown to reduce thermal sensation and improve perceived performance during exercise in hot environments, particularly when delivered via beverages or mouth rinses [14]. Its effects are mediated through activation of transient receptor potential melastatin 8 (TRPM8) receptors in the oral cavity, producing a cooling sensation [15,16]. However, a recent systematic review suggests that while menthol may improve heat tolerance and reduce perceived exertion, it may also impair thermoregulation and promote internal heat storage during prolonged exposure [17]. Despite these concerns, menthol delivery through beverages or rinses remains a practical and low-cost strategy that may complement hydration in settings where active cooling is limited [18].

Given the physiological challenges of firefighting in high-heat environments, recovery strategies should support fluid and electrolyte replenishment while enhancing thermal comfort. Menthol, which activates cold-sensing TRPM8 receptors and produces a cooling sensation, has gained attention as a perceptual cooling aid during and after exertion. Therefore, this study examined the effects of plain water, a carbohydrate–electrolyte solution, and a menthol-supplemented carbohydrate–electrolyte solution on hydration status, physiological recovery, and neuromuscular performance following simulated firefighting exercise. We hypothesised that electrolyte solutions would improve rehydration compared with water, and that menthol supplementation would help preserve explosive power.

## 2. Materials and methods

### 2.1. Participants

Twenty-four male operational firefighters (age: 32.8 ± 5.8 years) were recruited from a municipal fire department in Taiwan and voluntarily participated in this study. Only male firefighters were included to minimise variability related to sex-specific physiological responses. All participants were active-duty urban firefighters spanning a range of service ranks and operational roles. Individuals with a history of renal disease, metabolic disorders, or those taking medications known to affect fluid balance or thermoregulation were excluded. Exclusion criteria included musculoskeletal, cardiovascular, or neurological conditions that could interfere with safe participation in the performance assessments. Written informed consent was obtained from all participants prior to testing. The study protocol was reviewed and approved by the Institutional Review Board of Landseed International Hospital (IRB No. IRB-20-051-A2).

### 2.2. Experimental design and rehydration protocol

**Figure 1.**
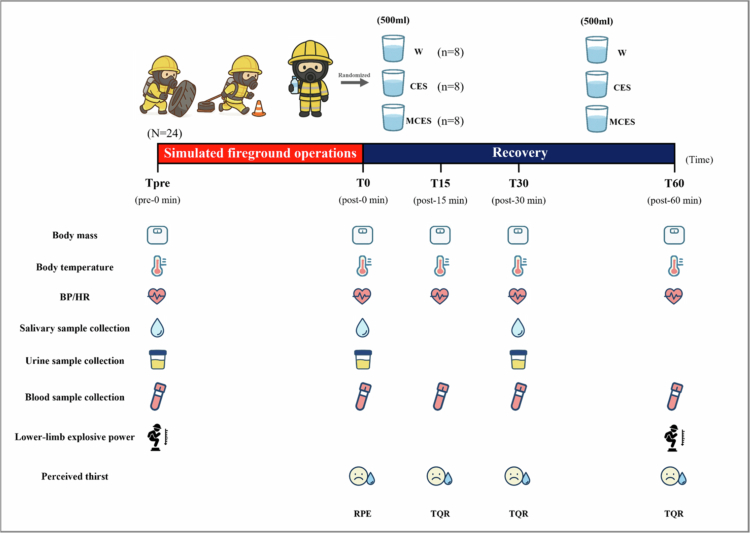
Experimental protocol. Abbreviations: W, water group; CES, carbohydrate-electrolyte solution group; MCES, menthol-supplemented carbohydrate-electrolyte solution group; BP, blood pressure; HR, heart rate; TQR, total quality recovery; RPE, rating of perceived exertion.

The overall experimental timeline and rehydration protocol are illustrated in Figure 1. Baseline assessments (Tpre) were conducted prior to the simulated firefighting exercise and included body weight, blood pressure, heart rate, forehead skin temperature, venous blood sampling, and lower-limb explosive strength (vertical jump test). Immediately after task completion (T0), participants removed their PPE and underwent the same physiological and perceptual evaluations.

Participants were randomly assigned to one of three rehydration conditions, with group allocation stratified by body weight to minimise inter-individual variation in fluid loss. The water group (W, *n* = 8) consumed plain water. The carbohydrate-electrolyte solution group (CES, *n* = 8) consumed a commercially available carbohydrate-electrolyte solution (Pocari Sweat®, Otsuka Pharmaceutical Co., Ltd; 122.2 kJ per 100 mL; 7.3% carbohydrate; sodium 21 mEq/L; potassium 5 mEq/L; chloride 16 mEq/L). The menthol-supplemented carbohydrate–electrolyte solution group (MCES, *n* = 8) received the same formulation with L-menthol added at a final concentration of 0.01%, a dosage previously shown to induce perceptual cooling and improve exercise performance in the heat [19].

The rehydration period lasted 60 minutes. Each participant consumed a total of 1.0 L of fluid, divided into two portions of 500 mL (T0-T30 and T30-T60). A fixed-volume rehydration approach was selected to standardise intake across groups and reflect operational constraints, rather than individualised sweat replacement. Physiological and perceptual measures were repeated at T30 and T60 to evaluate recovery responses.

### 2.3. Simulated occupational exercise

The simulated firefighting exercise consisted of a standardised protocol routinely implemented by the participating fire department. This protocol was designed to replicate real-world fireground demands and is widely used to evaluate physical readiness across domains including muscular strength, anaerobic and aerobic capacity, and functional agility.

Participants completed ten consecutive tasks while wearing full personal protective equipment and a self-contained breathing apparatus, with a total load of approximately 30 kg. The sequence included: (1) carrying two 25 kg foam concentrate drums for 50 m; (2) dragging a 78 kg, 22.5-inch tyre backward for 6 m; (3) dragging the same tyre forward for 6 m; (4) pulling a 12.6 kg, 15-inch tyre attached to a battling rope for 15 m; (5) completing five sets of over-and-under barrier manoeuvres (60 cm high, 190 cm apart); (6) flipping a 51 kg tyre 10 times; (7) striking a tyre 10 times with a 4.5 kg hammer; (8) dragging a 70 kg rescue dummy for 15 m; (9) hoisting a 9 kg hose vertically to a height of 5.9 m; and (10) ascending stairs from the second to the sixth floor while carrying two hoses (total 18 kg), then returning to the ground floor. All tasks conformed to assessment standards used in firefighter training and were completed under the supervision of certified fire instructors to ensure consistency and safety.

### 2.4. Body weight and hydration calculations

Body weight was assessed at five time points: prior to the simulated firefighting task (Tpre), immediately after task completion (T0), and during recovery at 15, 30, and 60 min (T15, T30, T60). Measurements were obtained using a multifrequency bioelectrical impedance analyser (InBody 570, Biospace Inc., Seoul, Korea). Participants were instructed to wear only underwear and to dry all sweat completely before each measurement. All assessments were conducted in a private room to ensure consistency and minimise variability.

At Tpre, baseline body composition parameters were also recorded to provide a comprehensive participant profile. These included fat mass, skeletal muscle mass, body fat percentage, and muscle mass percentage, as shown in Table 1. Subsequent time points (T0–T60) recorded only body weight for hydration tracking.

**Table 1.** Countermovement jump–derived lower-limb explosive power variables measured before the simulated firefighting task (Tpre) and after 60 min of recovery (T60).

Variable	Time	W	CES	MCES	ANOVA					
					Time		Group		Interaction	
					p	η2	p	η2	p	η2
**RFD 30 ms Max (N/Sec/Kg)**	Tpre	4.36 ± 1.41	4.21 ± 1.54	3.55 ± 1.71	0.430	0.030	0.291	0.111	0.811	0.020
	T60	4.26 ± 2.26	4.03 ± 2.20	2.87 ± 1.00						
**RFD (N/Sec/Kg)**	Tpre	5.58 ± 1.62	5.05 ± 1.32	4.46 ± 1.04	0.001^#^	0.407	0.469	0.469	0.329	0.101
	T60	4.72 ± 1.91	4.61 ± 1.23	4.11 ± 1.31						
**Time to force peak (Sec)**	Tpre	0.41 ± 0.09	0.44 ± 0.08	0.44 ± 0.09	0.013^#^	0.261	0.803	0.021	0.894	0.011
	T60	0.46 ± 0.12	0.47 ± 0.10	0.49 ± 0.13						
**Force peak (N)**	Tpre	1199 ± 212	1085 ± 79	1028 ± 177	0.027^#^	0.213	0.464	0.071	0.271	0.117
	T60	1087 ± 284*	1041 ± 73*	1011 ± 168						
**Relative force peak (N/Kg)**	Tpre	14.6 ± 2.76	13.2 ± 1.27	12.4 ± 1.47	0.033^#^	0.198	0.274	0.115	0.242	0.126
	T60	13.2 ± 2.80	12.8 ± 1.12*	12.2 ± 1.26						
Relative net impluse (N*Sec/Kg)	Tpre	0.94 ± 0.10	0.99 ± 0.10	0.99 ± 0.07	0.605	0.605	0.553	0.553	0.233	0.233
	T60	0.95 ± 0.14	0.95 ± 0.07	1.00 ± 0.06						
**Jump height (m)**	Tpre	0.37 ± 0.07	0.39 ± 0.06	0.34 ± 0.06	0.001^#^	0.435	0.579	0.051	0.061	0.233
	T60	0.33 ± 0.10*	0.36 ± 0.06*	0.34 ± 0.05						

Data are presented as mean ± SD (n = 8 per group). p values for group, time, and group × time interaction are from two-way repeated-measures ANOVA. * indicates a significant difference between Tpre and T60 within the same group (p < 0.05). # indicates a significant main effect of time, group, or interaction (p < 0.05). W, water group; CES, carbohydrate-electrolyte solution group; MCES, menthol-supplemented carbohydrate-electrolyte solution group. Non-significant interaction effects suggest that despite observed within-group changes, group-level recovery trajectories did not differ significantly.

Hydration status was evaluated based on changes in body weight across the experimental timeline. Fluid loss was calculated as the difference between Tpre and T0. Rehydration was assessed by comparing T60 body weight with Tpre. The dehydration rate was expressed as the percentage of body weight lost after exercise relative to baseline, whereas the rehydration rate reflected the proportion of fluid regained at T60 relative to the initial loss.

### 2.5. Basic physiological measurements

Physiological indicators, including forehead skin temperature, blood pressure, and heart rate, were assessed at five time points: before the simulated firefighting task (Tpre), immediately after task completion (T0), and at 15, 30, and 60 min post-task (T15, T30, T60). Forehead skin temperature was measured using a non-contact infra-red thermometer (FR 1DZ1, Microlife Corp., Taiwan) with participants seated in a stationary position. Blood pressure was measured using an automatic digital sphygmomanometer (HEM-7200, OMRON Healthcare Co., Ltd., Kyoto, Japan) in accordance with standard resting protocols. Heart rate was continuously monitored throughout the experiment using a validated chest-worn sensor (Polar H10, Polar Electro Oy, Kempele, Finland), and corresponding values were extracted for each designated time point. All physiological measurements were performed in a climate-controlled environment to minimise variability and ensure consistency across participants.

### 2.6. Blood sample collection and analysis

Venous blood samples were collected at four time points: before the simulated firefighting task (Tpre), immediately after task completion (T0), and at 30 and 60 min post-task (T30, T60). All blood draws were performed by a certified nurse using standard venipuncture procedures. Samples were immediately transported to the biochemical laboratory at the Graduate Institute of Sports Science, National Taiwan Sport University, for analysis.

Each whole blood sample was centrifuged at 3,000 rpm for 15 min at 4 °C using a refrigerated centrifuge to separate serum and plasma. Plasma osmolality (mOsm/kg H₂O) was measured using a freezing-point depression osmometer (Osmomat 3000; Gonotec GmbH, Berlin, Germany). Plasma electrolyte concentrations, including sodium (Na⁺) and potassium (K⁺), were determined with an automated biochemical analyser (Biolyte 2000 Autoanalyzer; Biocare, Lujhu Township, Taiwan) employing the ion-selective electrode method. Blood urea nitrogen (BUN), creatinine (CREA), and lactate concentrations were analysed using an automated biochemical analyser (Hitachi 7070 A; Hitachi High-Technologies Corp., Tokyo, Japan).

### 2.7. Saliva sample collection and analysis

Saliva samples were collected at three time points: before the simulated firefighting task (Tpre), immediately after task completion (T0), and 30 min post-task (T30). Participants were instructed to rinse their mouths with distilled water and wait for 1 min before sample collection to minimise contamination. Unstimulated whole saliva was then collected by passive drooling into sterile polypropylene tubes under resting conditions. Each sample was immediately placed on ice and subsequently analysed for salivary osmolality. Saliva osmolality (mOsm/kg H₂O) was measured using a freezing-point depression osmometer (Osmomat 3000; Gonotec GmbH, Berlin, Germany).

### 2.8. Urine sample collection and analysis

Urine samples were collected at three time points: before the simulated firefighting task (Tpre), and at immediately post-task (T0) and 30 min post-task (T30). Participants provided midstream urine samples in sterile medical-grade collection cups. All samples were promptly analysed to assess hydration status and urinary electrolyte concentrations.

Urine specific gravity (USG) was measured using reagent strip tests (URS-10; Teco Diagnostics, Anaheim, CA, USA) and analysed with a semi-automated urine chemistry reader (Uritek TC-101; Teco Diagnostics, Anaheim, CA, USA). USG values were classified into discrete levels (1.000, 1.005, 1.010, 1.015, 1.020, 1.025, 1.030).

Urine colour was evaluated visually using an eight-point scale [20], with assessments conducted under standardised lighting conditions by the same investigator to minimise bias.

Urine osmolality (mOsm/kg H₂O) was determined using a freezing-point depression osmometer (Osmomat 3000; Gonotec GmbH, Berlin, Germany). Urinary electrolyte concentrations (Na⁺ and K⁺) were measured with the ion-selective electrode method as described for plasma analysis.

### 2.9. Perceptual measures of exertion, thirst, and recovery

Perceived exertion was assessed immediately after the simulated firefighting task (T0) using the Borg rating of perceived exertion (RPE) scale, which ranges from 6 to 20, with higher scores indicating greater subjective effort. Participants reported their perceived exertion at this single post-task assessment to evaluate the acute physical strain induced by the task.

Thirst sensation was evaluated using a visual analogue scale (VAS), consisting of a 10-cm horizontal line anchored by “not thirsty at all” on the left and “extremely thirsty” on the right. This format, adapted from validated versions [21], required participants to mark the point that best reflected their subjective perception of thirst. The distance from the left end to the mark was measured in millimetres and recorded for analysis. Thirst ratings were collected at four time points: immediately post-task (T0) and at 15, 30, and 60 min of recovery (T15, T30, T60), enabling comparisons of rehydration responses among groups.

Subjective recovery status was evaluated using the Total Quality Recovery (TQR) scale, ranging from 6 (very poor) to 20 (very good), with higher values indicating better recovery perception [22]. Participants reported TQR scores at T15, T30, and T60 to monitor short-term recovery progression and compare the effectiveness of the different rehydration strategies.

### 2.10. Lower-limb explosive power assessment

Lower-limb explosive power was assessed using a portable force plate (Kistler 9260AA, Kistler Ltd., Switzerland) at two time points: before the simulated firefighting task (Tpre) and after 60 min of recovery (T60). Participants performed three maximal countermovement jumps with 1-min rest intervals between attempts. Each jump began from a standardised half-squat position with knees flexed to approximately 90°, while hands were kept on the hips to eliminate arm swing. An upright trunk posture was maintained during take-off. All trials were conducted in a private setting under the supervision of trained investigators to ensure reliability.

Force–time data were collected at a sampling rate of 1000 Hz and analysed using the manufacturer’s software. Primary outcome variables included peak ground reaction force, time to peak force, rate of force development at 30 ms, net impulse, relative force normalised to body mass, and jump height. These parameters were used to evaluate neuromuscular performance and explosive strength capacity under pre- and post-task hydration conditions.

### 2.11. Statistical analysis

All analyses were conducted using SPSS Statistics v.25.0 (IBM Corp., Armonk, NY, USA). One-way analysis of variance (ANOVA) was performed to examine baseline differences among the three experimental groups (W, SS, MSS). For outcome variables measured at multiple time points, repeated-measures ANOVA was applied to evaluate the main effects of time, group, and the time × group interaction. For variables with only two time points, including lower-limb explosive power (Table 1), a two-way mixed-design ANOVA was used, with time as the within-subject factor and group as the between-subject factor. Paired-samples t-tests were conducted within groups to compare pre- and post-intervention values in Table 1. For single-time-point variables and change scores, one-way ANOVA was used to assess group differences. When significant main effects or interactions were detected, Bonferroni-adjusted post hoc tests were performed to identify pairwise differences. Statistical significance was set at *p* < 0.05, and all data are presented as mean ± standard deviation (SD).

## 3. Result

### 3.1. Participant characteristics

All participants were healthy male firefighters who met the inclusion criteria for this study. Baseline anthropometric and body composition data are provided in Supplementary Table S1. There were no significant baseline differences among the three groups in age, height, body mass, body mass index (BMI), fat mass, body fat percentage, muscle mass, or muscle mass percentage (all *p* > 0.05), indicating that the groups were comparable prior to the intervention.

### 3.2. Completion time and peak heart rate

All participants successfully completed the standardised simulated firefighting protocol while wearing full PPE and a self-contained breathing apparatus. Total task completion time and peak heart rate at T0 did not differ significantly among groups (Supplementary Figure S1; all *p* > 0.05).

### 3.3. Changes in body weight and hydration status

**Figure 2.**
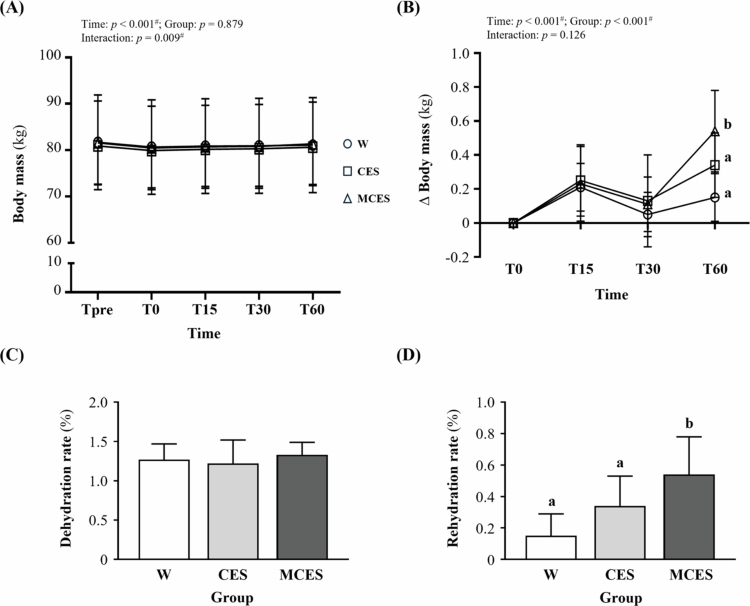
Changes in (A) body mass, (B) *Δ* body mass, (C) dehydration rate, and (D) rehydration rate during the 60-min recovery period following the simulated task across the W, CES, and MCES groups. Data are presented as mean ± SD (*n* = 8 per group). # indicates a significant main effect of time by repeated-measures ANOVA (*p* < 0.05). Different superscript letters (a, b) indicate statistically significant differences between groups (*p* < 0.05). W, water group; CES, carbohydrate-electrolyte solution group; MCES, menthol-supplemented carbohydrate-electrolyte solution group.

As shown in Figure 2B, net body mass change also exhibited a significant main effect of time (F(1.86, 39.01) = 15.27, *p* < 0.001, η² = 0.421) and group (F(2, 21) = 12.63, *p* < 0.001, η² = 0.546), while the time × group interaction was not significant (F(3.72, 39.01) = 1.95, *p* = 0.126, η² = 0.157). Post hoc comparisons showed that the MCES group had greater body mass restoration than both W (*p* = 0.013) and the CES group (*p* < 0.001). At T60, group differences were significant (F(2, 21) = 7.91, *p* = 0.003, η² = 0.430), and post hoc analysis confirmed that the MCES group achieved significantly greater body mass restoration than the W group (*p* = 0.002).

Dehydration and rehydration rates during the recovery period are shown in Figure 2C and D. Dehydration rate did not differ among groups (F(2, 21) = 0.42, *p* = 0.660, η² = 0.039), indicating that body mass loss immediately after task completion was comparable across groups. In contrast, rehydration rate differed significantly (F(2, 21) = 11.41, *p* < 0.001, η² = 0.521), and post hoc analysis revealed that the W group had significantly lower rehydration rates compared to the CES group (*p* = 0.016) and the MCES group (*p* < 0.001).

### 3.4. Physiological recovery responses

**Figure 3.**
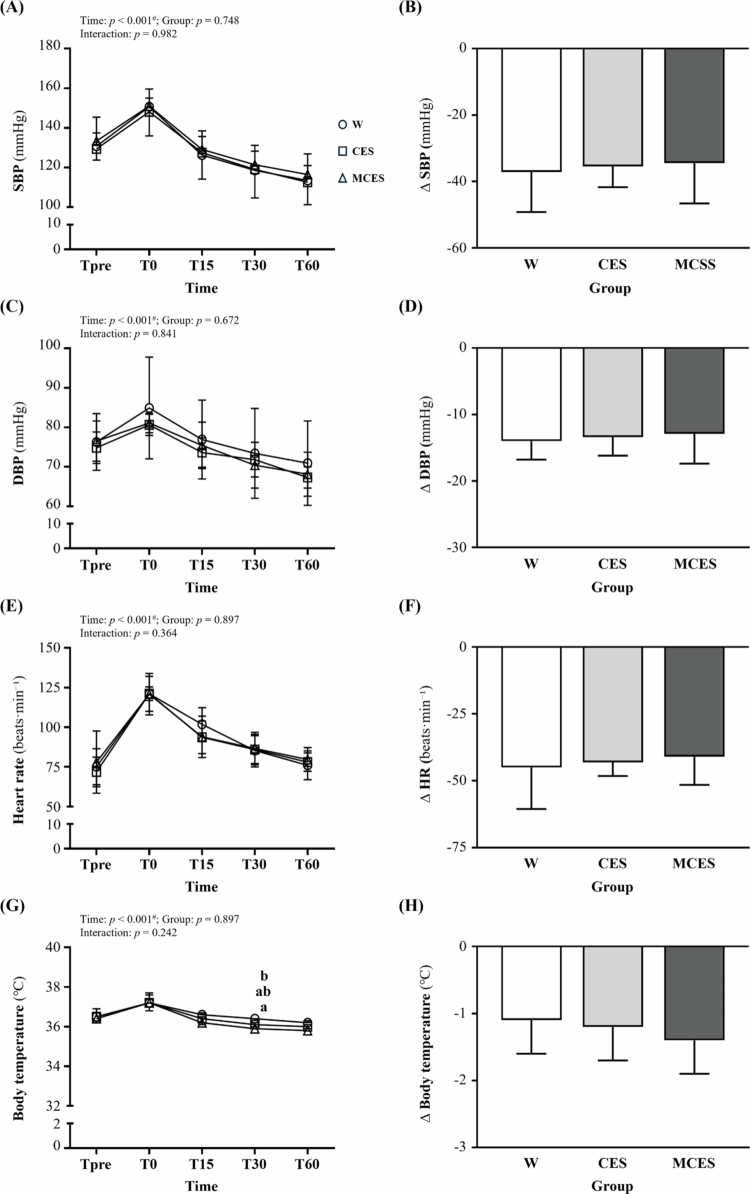
Changes in (A) systolic blood pressure (SBP), (B) ΔSBP, (C) diastolic blood pressure (DBP), (D) ΔDBP, (E) heart rate (HR), (F) ΔHR, (G) body temperature, and (H) *Δ* body temperature during the 60-min recovery period across the W, CES, and MCES groups. Data are presented as mean ± SD (*n* = 8 per group). # indicates a significant main effect of time by repeated-measures ANOVA (*p* < 0.05). Different superscript letters (a, b) indicate statistically significant differences between groups (*p* < 0.05). *Δ* values represent absolute changes from Tpre and are expressed in original units (mmHg, beats·min⁻¹, °C). W, water group; CES, carbohydrate-electrolyte solution group; MCES, menthol-supplemented carbohydrate-electrolyte solution group; SBP, systolic blood pressure; DBP, diastolic blood pressure; HR, heart rate.

Figure 3A shows systolic blood pressure (SBP) changes during recovery. A significant main effect of time was observed (F(2.22, 46.63) = 89.50, *p* < 0.001, η² = 0.810), whereas no group effect or interaction was detected (*p* > 0.05), indicating similar SBP patterns among groups. Corresponding ∆SBP (Figure 3B) also showed no significant group differences (*p* = 0.875).

Diastolic blood pressure (DBP) (Figure 3C) demonstrated a main effect of time (F(2.43, 51.02) = 68.58, *p* < 0.001, η² = 0.766), with no group or interaction effects (*p* > 0.05). Similarly, ∆DBP (Figure 3D) did not differ across groups (*p* = 0.827).

Heart rate (HR) (Figure 3E) also showed a main effect of time (F(2.67, 56.15) = 153.36, *p* < 0.001, η² = 0.880), while group and interaction effects were non-significant (*p* > 0.05). Consistently, ∆HR (Figure 3F) showed no group differences (*p* = 0.780).

Body temperature (Figure 3G) exhibited a significant time effect (F(2.45, 51.44) = 73.57, *p* < 0.001, η² = 0.778). Although overall group and interaction effects were not significant, a difference was detected at T30 (F(2, 21) = 3.85, *p* = 0.038, η² = 0.268), with MCES higher than W (*p* = 0.040). However, ∆body temperature (Figure 3H) showed no group differences (*p* = 0.299).

### 3.5. Blood and salivary biochemical responses

**Figure 4.**
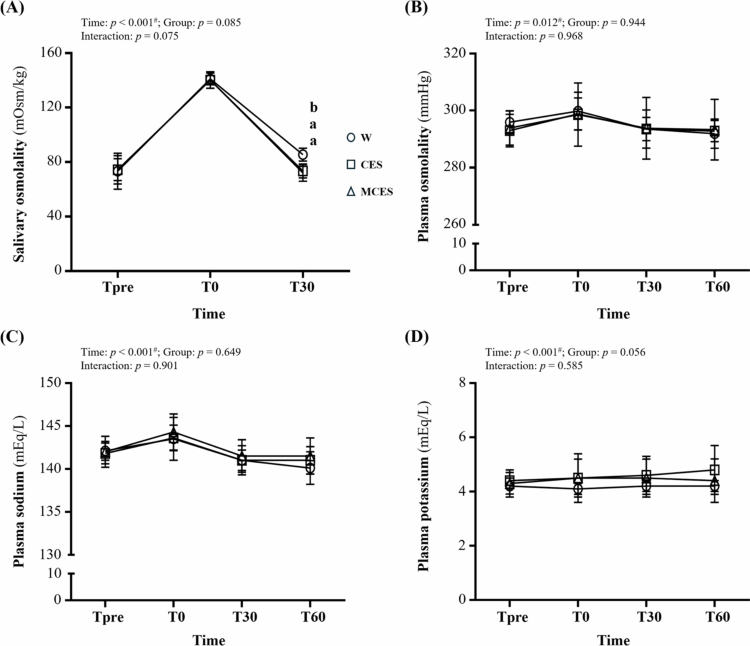
Changes in (A) salivary osmolality, (B) plasma osmolality, (C) plasma sodium concentration, and (D) plasma potassium concentration during the 60-min recovery period across the W, CES, and MCES groups. Data are presented as mean ± SD (*n* = 8 per group). # indicates a significant main effect of time by repeated-measures ANOVA (*p* < 0.05). Different superscript letters (a, b) indicate statistically significant differences between groups (*p* < 0.05). W, water group; CES, carbohydrate-electrolyte solution group; MCES, menthol-supplemented carbohydrate-electrolyte solution group.

Salivary osmolality (Figure 4A) showed a main effect of time (F(1.40, 29.35) = 588.93, *p* < 0.001, η² = 0.966) with no overall group or interaction effects. At T30, the W group exhibited significantly higher values than the CES group (*p* = 0.001) and the MCES group (*p* < 0.001).

Plasma osmolality (Figure 4B) demonstrated a time effect (F(2.23, 46.84) = 4.66, *p* = 0.012, η² = 0.181), with no significant group or interaction effects.

Plasma sodium (Figure 4C) varied significantly over time (F(2.03, 42.70) = 69222.53, *p* < 0.001, η² = 1.000), while group and interaction effects were not significant.

Plasma potassium (Figure 4D) also showed a time effect (F(1.10, 23.08) = 93264.91, *p* < 0.001, η² = 1.000), with no significant group or interaction effects.

**Figure 5.**
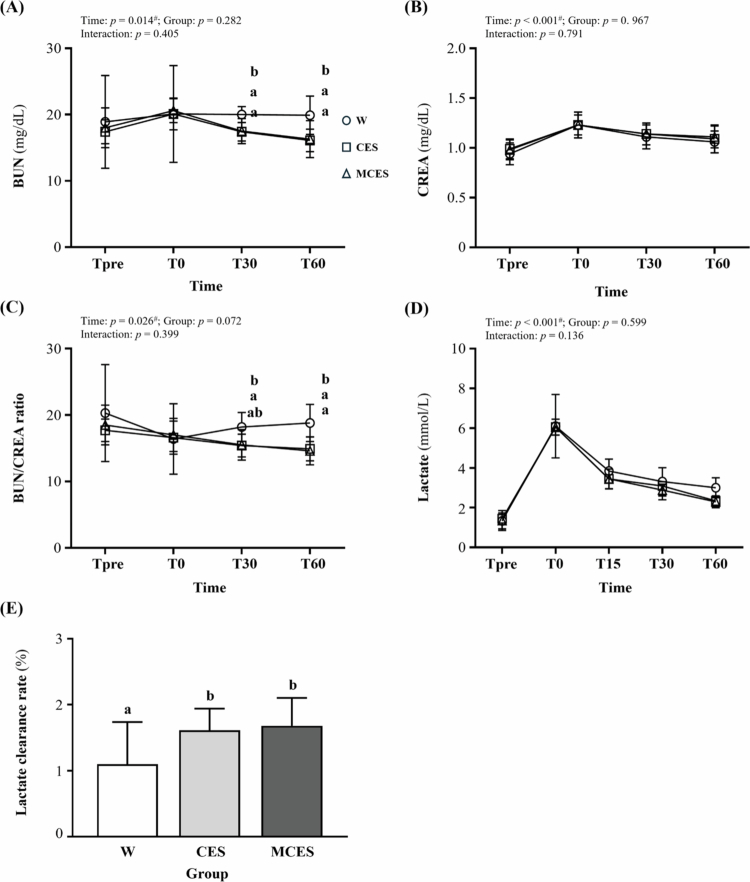
Changes in (A) blood urea nitrogen (BUN), (B) creatinine (CREA), (C) BUN/CREA ratio, (D) blood lactate concentration, and (E) lactate clearance rate from pre-task (Tpre) and immediately post-task (T0) to 30 and 60 min of recovery across the W, CES, and MCES groups. Data are presented as mean ± SD (*n* = 8 per group). # indicates a significant main effect of time by repeated-measures ANOVA (*p* < 0.05). Different superscript letters (a, b) indicate statistically significant differences between groups at the same time point (*p* < 0.05). W, water group; CES, carbohydrate-electrolyte solution group; MCES, menthol-supplemented carbohydrate-electrolyte solution group; BUN, blood urea nitrogen; CREA, creatinine.

BUN (Figure 5A) showed a time effect (F(1.95, 40.93) = 4.78, *p* = 0.014, η² = 0.185). At T30 and T60, the W group had significantly higher concentrations than both the CES group (*p* = 0.008 and 0.020, respectively) and the MCES group (*p* = 0.012 and 0.026, respectively).

Creatinine (Figure 5B) varied by time (F(3, 63) = 41.71, *p* < 0.001, η² = 0.665), with no significant group or interaction effects.

The BUN/CREA ratio (Figure 5C) showed a time effect (F(2.06, 43.16) = 3.93, *p* = 0.026, η² = 0.158). At T30, the W group had a significantly higher ratio than the CES group (*p* = 0.044). At T60, the W group exhibited a significantly higher ratio than both the CES group (*p* = 0.008) and the MCES group (*p* = 0.004).

Lactate concentration (Figure 5D) demonstrated a time effect (F(2.43, 50.99) = 209.76, *p* < 0.001, η² = 0.909), with no significant group or interaction effects.

Lactate clearance (Figure 5E) showed a group effect (F(2, 21) = 3.48, *p* = 0.049, η² = 0.249). Post hoc analysis indicated that the W group had a significantly lower clearance rate than the CES group (*p* = 0.007) and the MCES group (*p* = 0.004).

### 3.6. Urinary markers of hydration and electrolyte balance

**Figure 6.**
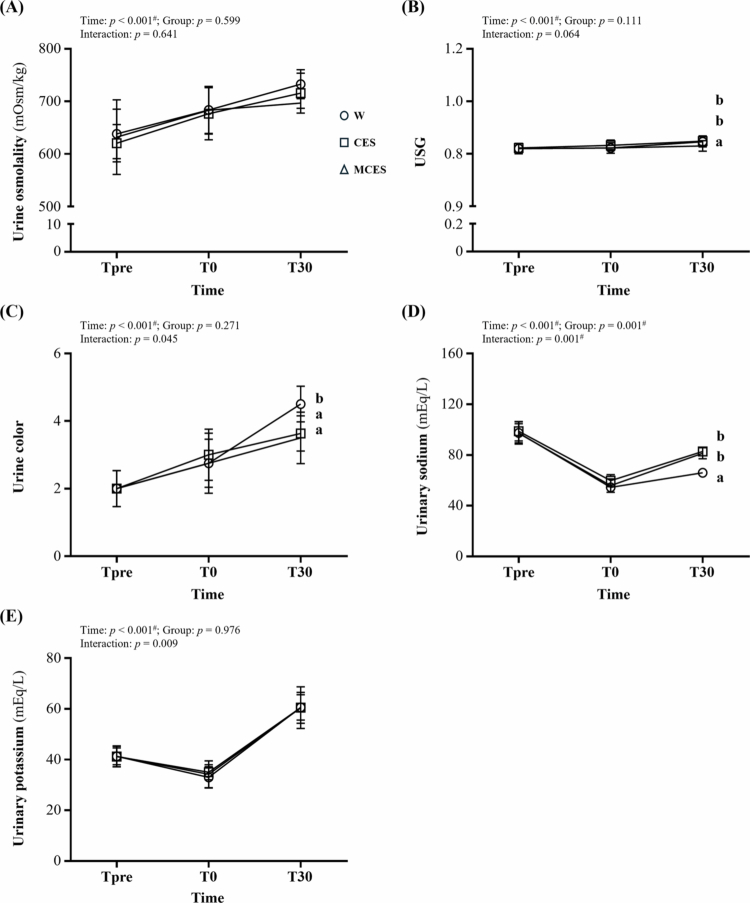
Changes in (A) urine osmolality, (B) urine specific gravity (USG), (C) urine colour, (D) urinary sodium concentration, and (E) urinary potassium concentration from Tpre and T0 to 30 min of recovery (T30) across the W, CES, and MCES groups. Data are presented as mean ± SD (*n* = 8 per group). # indicates a significant main effect of time by repeated-measures ANOVA (*p* < 0.05). Different superscript letters (a, b) indicate statistically significant differences between groups at the same time point (*p* < 0.05). W, water group; CES, carbohydrate-electrolyte solution group; MCES, menthol-supplemented carbohydrate-electrolyte solution group; USG, Urine specific gravity.

Urine osmolality (Figure 6A) demonstrated a significant effect of time (F(2, 42) = 31.49, *p* < 0.001, η² = 0.600), whereas the group effect and time × group interaction were not significant, indicating similar response patterns across conditions.

Urine specific gravity (USG; Figure 6B) showed a time effect (F(2, 42) = 29.17, *p* < 0.001, η² = 0.581) but no overall group effect. The time × group interaction approached significance (*p* = 0.064). At T30, the MCES group exhibited significantly lower USG values than both the W group (*p* = 0.027) and the CES group (*p* = 0.010).

Urine colour (Figure 6C) showed significant effects of time (*p* < 0.001), group (*p* = 0.007), and the time × group interaction (*p* = 0.045). At T30, the W group exhibited higher urine colour scores than both the CES group (*p* = 0.028) and the MCES group (*p* = 0.011).

Urinary sodium concentration (Figure 6D) demonstrated time (*p* < 0.001), group (*p* = 0.001), and time × group interaction (*p* = 0.001) effects. At T30, the W group had significantly lower sodium concentrations than the CES group (*p* < 0.001) and the MCES group (*p* = 0.012).

Urinary potassium concentration (Figure 6E) showed a time effect (F(2, 42) = 182.20, *p* < 0.001, η² = 0.897) with no group or interaction effects, indicating that potassium followed a similar temporal pattern across all groups.

### 3.7. Perceptual responses to thirst and fatigue

Perceived exertion (RPE scale) did not differ significantly between the W, CES, and MCES groups immediately after the simulated firefighting task (T0) (*p* > 0.05), as shown in Supplementary Figure S2.

**Figure 7.**
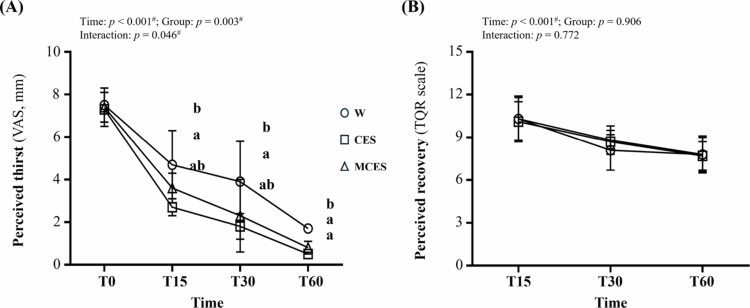
Changes in (A) perceived thirst (visual analogue scale, VAS) and (B) perceived recovery (TQR scale) during the 60-min recovery period across the W, CES, and MCES groups. Data are presented as mean ± SD (*n* = 8 per group). # indicates a significant main effect of time by repeated-measures ANOVA (*p* < 0.05). Different superscript letters (a, b) indicate statistically significant differences between groups (*p* < 0.05). W, water group; CES, carbohydrate-electrolyte solution group; MCES, menthol-supplemented carbohydrate-electrolyte solution group; VAS, thirst visual analogue scale; TQR, total quality recovery.

Perceived thirst (VAS; Figure 7A) demonstrated significant effects of time (F(1.84, 38.72) = 265.54, *p* < 0.001, η² = 0.927), group (F(2, 21) = 7.75, *p* = 0.003, η² = 0.425), and time × group interaction (F(3.69, 38.72) = 2.75, *p* = 0.046, η² = 0.207). Post hoc comparisons showed that participants in the water group reported greater thirst than those in the CES group (*p* = 0.013) and the MCES group (*p* < 0.001). At T15, the water group reported significantly higher thirst scores than the CES group (*p* = 0.003). At T30, thirst scores in the water group remained higher than in the CES group (*p* = 0.027). At T60, differences were most pronounced, with the water group reporting significantly higher thirst ratings than both the CES (*p* < 0.001) and MCES groups (*p* < 0.001).

Perceived recovery (TQR; Figure 7B) demonstrated a significant effect of time (F(1.47, 30.94) = 50.02, *p* < 0.001, η² = 0.704), whereas group (*p* = 0.906) and time × group interaction (*p* = 0.772) were not significant. These results indicate that recovery perception improved progressively over time, with no meaningful differences between rehydration conditions.

### 3.8. Recovery of lower-limb explosive power

Countermovement jump variables at Tpre and T60 are summarised in Table 1. A main effect of time was observed for rate of force development (RFD) (F(1, 21) = 14.43, *p* = 0.001, η² = 0.407), time to force peak (F(1, 21) = 7.43, *p* = 0.013, η² = 0.261), force peak (F(1, 21) = 5.68, *p* = 0.027, η² = 0.213), relative force peak (F(1, 21) = 5.19, *p* = 0.033, η² = 0.198), and jump height (F(1, 21) = 16.20, *p* = 0.001, η² = 0.435). Group and interaction effects were not significant (all *p* > 0.05).

Within-group comparisons revealed that in the water group, force peak (*p* = 0.034) and jump height (*p* = 0.036) declined significantly from Tpre to T60. In the CES group, reductions were observed in force peak (*p* = 0.018), relative force peak (*p* = 0.038), and jump height (*p* = 0.003). In contrast, no significant changes were detected in the MCES group (all *p* > 0.05).

## 4. Discussion

Adequate rehydration following intense physical exertion in high temperature environments is critical for restoring physiological homoeostasis and maintaining performance capacity. Fire suppression performed under high thermal stress in full PPE produces substantial fluid and electrolyte losses that lead to measurable dehydration and physiological strain [13]. In the present study, carbohydrate-electrolyte solutions with and without L-menthol achieved greater rehydration than water during the 60-minute post-task period, reflected by larger restoration of body mass and more favourable hydration indices. These findings align with evidence that fluids containing sodium, potassium, and carbohydrate enhance fluid retention, stimulate voluntary intake, and accelerate restoration of fluid balance compared with water [23]. Operational guidance for heat-exposed occupational and athletic settings, including firefighters, recommends sodium-containing fluids to help maintain fluid balance and reduce heat strain during work cycles [10,24]. Current evidence therefore supports prioritising electrolyte-containing solutions for rapid recovery following simulated firefighting tasks.

To emulate real rescue operations, participants consumed the assigned fluids during the 60-minute post-task recovery period after the simulated firefighting task. Specifically, body mass restoration showed a group effect driven by the menthol electrolyte condition (MCES), which exceeded both water and the standard carbohydrate–electrolyte solution (CES) during the recovery period. Rehydration rate was higher for both electrolyte fluids than for water. Electrolyte fluids were also associated with urinary profiles consistent with enhanced fluid retention, including higher urinary sodium and lower urine colour, with between-group differences most evident at T30, and lower salivary osmolality at T30 in both electrolyte conditions versus water, consistent with more effective restoration of body water. At T30 urine specific gravity was lower in the MCES group than in W and CES group, which supports an early advantage for the menthol electrolyte fluid on fluid conservation. Under heat stress, higher sodium concentrations in ingested fluids help maintain plasma sodium and plasma volume with matched fluid intake, and personalised sodium-containing strategies improve fluid balance [25,26]. In simulated firefighting, Tabuchi et al. reported that ice slurry before work reduced thermal strain and that a sodium-containing carbohydrate fluid after work accelerated recovery of fluid balance, reflected by favourable changes in serum sodium and osmolality [27]. A randomised crossover trial also showed greater fluid retention and lower early urine output with sodium-containing fluids over several hours versus water [28]. Our results accord with this pattern, as electrolyte fluids produced more favourable urinary and salivary hydration markers than water, and the greater net body mass restoration was driven by the MCES group.

Renal and metabolic indices in our dataset tracked the early hydration responses.At 30 and 60 minutes, the water group showed higher BUN and a higher BUN to creatinine ratio with similar creatinine, which is consistent with a prerenal hypovolemic pattern after heat exposure. This aligns with faster rehydration in the electrolyte conditions. Under matched fluid intake, sodium-containing oral rehydration solutions reduce cumulative urine output and yield a more positive net fluid and sodium balance, indicating more effective early fluid retention [29]. Consistent with this, operational reviews in wildland firefighters emphasise regular fluid and electrolyte intake during suppression [30]. Cardiovascular and thermal measures changed markedly across the first hour of recovery as PPE was removed and fluids were consumed. Systolic and diastolic blood pressure and heart rate showed large time effects with no group differences, and change scores were comparable across drinks. Body temperature declined over time with no overall group effect, and the transient difference at 30 minutes with the menthol condition higher than water did not persist. Together, these patterns indicate that early cardiovascular recovery was dominated by unloading and heat loss as gear was removed and fluids were consumed, while beverage formulation made little immediate difference in heart rate or blood pressure [12,28].

During recovery, VAS thirst scores were lower after the electrolyte solution than after water, with the clearest separation at 30 minutes, and the menthol fluids showed no consistent additional reduction beyond the standard electrolyte solution. Perceived recovery improved over time with no between-group differences, indicating that the composition of the ingested fluids did not alter global recovery perception within the first hour. This finding is consistent with previous research showing that a carbohydrate drink containing menthol does not improve thermal perception or exercise capacity [31]. In contrast, oral L-menthol ingestion during exercise improved breathing comfort and extended time to exhaustion in well trained runners [32]. Consistent with that study, our cardiovascular and thermal indices and perceptual measures did not differ between fluids. The only divergence was in performance, with the menthol electrolyte fluid maintained short duration high power from Tpre to T60 while water and the standard electrolyte fluid showed within group declines. Within groups, peak force and jump height declined from pre to post with water and with the standard electrolyte solution, whereas no pre to post decrements were detected with L-menthol, indicating maintenance rather than superiority and aligning with evidence that heat stress can reduce neuromuscular output through central and peripheral pathways, so easing thermal discomfort may help preserve central drive for brief high power tasks [6,33]. Trials in the heat show that oral L-menthol reduces thermal sensation and supports performance, reflected by higher work rate, longer time to exhaustion, and preservation of short-duration power across controlled cycling protocols [34,35]. Our data align with this profile, as cardiovascular indices and plasma osmolality did not differ between groups, rehydration was comparable between the menthol and standard carbohydrate–electrolyte solution, and menthol was associated with maintenance of brief high-power output during recovery. Oral menthol exposure in the heat increased work rate and extended time to exhaustion while mean body temperature remained unchanged or elevated, indicating performance support without physiological cooling [18]. Followup work in hot conditions reinforced that menthol mouth-rinsing can support performance without consistent reductions in core temperature, consistent with modulation of thermal sensation and perceived effort rather than systemic cooling [36].

Menthol activates TRPM8 channels on oral and oropharyngeal sensory neurons and generates a trigeminally mediated cooling signal that recalibrates thermal sensation and perceived effort during heat exposure. These sensory effects are often observed without parallel reductions in core temperature [37]. In the heat, meta-analytic evidence shows that menthol mouth rinsing yields modest and consistent improvements in exercise capacity and thermal comfort, typically accompanied by lower thermal sensation and perceived exertion with little change in core temperature or cardiovascular variables, with effects most evident when menthol is delivered during exercise and variation in task intensity, environmental heat load, and delivery parameters pointing to a predominantly perceptual pathway rather than enhanced heat loss [17]. The review recommends pairing menthol use with sound hydration practice rather than relying on perceptual cooling alone, as menthol may alter thermal perception without improving heat dissipation [17]. An expert consensus in heat physiology and performance recommends menthol as a perceptual cooling aid for athletes preparing for and competing in hot environments, to be used alongside comprehensive heat management practices [38]. To emulate real rescue operations, participants consumed the assigned fluids during the post-task recovery period following the simulated firefighting task. In our recovery context the menthol electrolyte formulation maintained short-duration high-power output from Tpre to T60, whereas water and the standard electrolyte condition showed within-group declines, which is consistent with perceptual relief rather than systemic cooling.

## 5. Limitations

This study was conducted under applied field conditions involving professional firefighters, where operational demands and physiological load are inherently variable. Consequently, the study design prioritised feasibility and ecological validity, and a parallel-group approach was employed. The sample size was based on the number of eligible professional firefighters who consented to participate, with consideration given to operational schedules and participant safety. In addition, only male firefighters were included in this study. This was partly due to the low proportion of female firefighters within the participating fire departments, which limited their recruitment. Furthermore, restricting the sample to a single sex helped reduce variability associated with sex-specific physiological differences, particularly in thermoregulation and fluid balance. While this design enhances feasibility under real-world occupational conditions, it may limit the detection of subtle between-group physiological differences. Future research with larger cohorts is recommended to enhance the generalisability of these findings.

## 6. Conclusion

In simulated occupational exercise performed in full PPE, sodium-containing carbohydrate solutions enhanced early recovery relative to water. During the 60-min post-task period, both electrolyte solutions (CES and MCES) facilitated greater body mass restoration, more favourable renal and urinary hydration indices, lower perceived thirst, and faster lactate clearance than water. Cardiovascular and thermal indices returned toward baseline over time with similar trajectories across groups, suggesting that passive recovery and unloading rather than beverage composition were the primary drivers of these responses. The MCES solution did not provide additional hydration or thirst benefits compared with CES, but preserved jump performance and peak force, supporting a perceptual adjunct role under PPE-induced thermal strain. Taken together, sodium-containing carbohydrate solutions should be prioritised in the first hour of recovery to restore body water and support metabolic recovery, whereas menthol supplementation may serve as an adjunct to improve comfort and help maintain high-intensity performance capacity, thereby supporting both hydration and performance recovery in heat-stressed occupational settings.

## Supplementary Material

Supplementary Material SUPPLEMENTARY FIGURE TABLE

## Data Availability

The data presented in this study are available within the article.

## Publication statement

This manuscript has not been published elsewhere and has not been submitted simultaneously for publication elsewhere.

## References

[1] Kollock R, Thomas J, Hale D, et al. The effects of firefighter equipment and gear on the static and dynamic postural stability of fire cadets. Gait Posture. 2021;88:292–296. doi: 10.1016/j.gaitpost.2021.05.034 34153807

[2] Park H, Park J, Lin S-H, et al. Assessment of firefighters’ needs for personal protective equipment. Fashion and Textiles. 2014;1(1):8. doi: 10.1186/s40691-014-0008-3

[3] Ghiyasi S, Nabizadeh H, Jazari MD, et al. The effect of personal protective equipment on thermal stress: an experimental study on firefighters. Work. 2020;67(1):141–147. doi: 10.3233/WOR-203259 32955479

[4] Pearson SJ, Highlands B, Jones R, et al. Comparisons of core temperature between a telemetric pill and heart rate estimated core temperature in firefighters. Saf Health Work. 2022;13(1):99–103. doi: 10.1016/j.shaw.2021.11.003 35936211 PMC9346945

[5] Games KE, Winkelmann ZK, McGinnis KD, et al. Functional performance of firefighters after exposure to environmental conditions and exercise. J Athl Train. 2020;55(1):71–79. doi: 10.4085/1062-6050-75-18 31876454 PMC6961651

[6] Nybo L, Rasmussen P, Sawka MN. Performance in the heat-physiological factors of importance for hyperthermia-induced fatigue. Compr Physiol. 2014;4(2):657–689. doi: 10.1002/j.2040-4603.2014.tb00554.x 24715563

[7] Fullagar H, Notley SR, Fransen J, et al. Cooling strategies for firefighters: effects on physiological, physical, and visuo-motor outcomes following fire-fighting tasks in the heat. J Therm Biol. 2022;106:103236. doi: 10.1016/j.jtherbio.2022.103236 35636886

[8] Gonzalez DE, Lanham SN, Martin SE, et al. Firefighter health: a narrative review of occupational threats and countermeasures. Healthcare (Basel). 2024;12(4):440. doi: 10.3390/healthcare12040440 38391814 PMC10888326

[9] Franklin BA, Eijsvogels TMH, Pandey A, et al. Physical activity, cardiorespiratory fitness, and cardiovascular health: a clinical practice statement of the ASPC part I: bioenergetics, contemporary physical activity recommendations, benefits, risks, extreme exercise regimens, potential maladaptations. Am J Prev Cardiol. 2022;12:100424. doi: 10.1016/j.ajpc.2022.100424 36281324 PMC9586848

[10] Holland-Winkler AM, Hamil BK. Hydration considerations to improve the physical performance and health of firefighters. J Funct Morphol Kinesiol. 2024;9(4):182. doi: 10.3390/jfmk9040182 39449476 PMC11503342

[11] Evans GH, James LJ, Shirreffs SM, et al. Optimizing the restoration and maintenance of fluid balance after exercise-induced dehydration. J Appl Physiol (1985). 2017;122(4):945–951. doi: 10.1152/japplphysiol.00745.2016 28126906

[12] Périard JD, Eijsvogels TMH, Daanen HAM. Exercise under heat stress: thermoregulation, hydration, performance implications, and mitigation strategies. Physiol Rev. 2021;101(4):1873–1979. doi: 10.1152/physrev.00038.2020 33829868

[13] Wohlgemuth K, Sekiguchi Y, Mota J. Overexertion and heat stress in the fire service: a new conceptual framework. Am J Ind Med. 2023;66(8):705–709. doi: 10.1002/ajim.23482 37062940

[14] Jeffries O, Waldron M. The effects of menthol on exercise performance and thermal sensation: a meta-analysis. J Sci Med Sport. 2019;22(6):707–715. doi: 10.1016/j.jsams.2018.12.002 30554924

[15] Yin Y, Lee SY. Current view of ligand and lipid recognition by the menthol receptor TRPM8. Trends Biochem Sci. 2020;45(9):806–819. doi: 10.1016/j.tibs.2020.05.008 32532587 PMC9284947

[16] Gavel EH, Hawke KV, Bentley DJ, et al. Menthol mouth rinsing is more than just a mouth wash-swilling of menthol to improve physiological performance. Front Nutr. 2021;8:691695. doi: 10.3389/fnut.2021.691695 34307438 PMC8292615

[17] Gavel EH, Barreto G, Hawke KV, et al. How cool is that? The effects of menthol mouth rinsing on exercise capacity and performance: a systematic review and meta-analysis. Sports Med Open. 2024;10(1):18. doi: 10.1186/s40798-024-00679-8 38381237 PMC10881929

[18] Jeffries O, Goldsmith M, Waldron M. L-Menthol mouth rinse or ice slurry ingestion during the latter stages of exercise in the heat provide a novel stimulus to enhance performance despite elevation in mean body temperature. Eur J Appl Physiol. 2018;118(11):2435–2442. doi: 10.1007/s00421-018-3970-4 30128853 PMC6182327

[19] Mündel T, Jones DA. The effects of swilling an L(-)-menthol solution during exercise in the heat. Eur J Appl Physiol. 2010;109(1):59–65. doi: 10.1007/s00421-009-1180-9 19727797

[20] Armstrong LE, Maresh CM, Castellani JW, et al. Urinary indices of hydration status. Int J Sport Nutr. 1994;4(3):265–279. doi: 10.1123/ijsn.4.3.265 7987361

[21] 2019 Matias A, Dudar M, Kauzlaric J, et al. Rehydrating efficacy of maple water after exercise-induced dehydration. J Int Soc Sports Nutr. 2019;16(1):5. doi: 10.1 186/s12970-019-0273-z. 10.1186/s12970-019-0273-z PMC6371469 30744654

[22] Coope OC, Reales Salguero A, Spurr T, et al. Effects of Root Extract of Ashwagandha (Withania somnifera) on Perception of Recovery and Muscle Strength in Female Athletes. Eur J Sport Sci. 2025; 25(3): e12265. doi:10.1 002/ejsc.12265. 10.1002/ejsc.12265 PMC11829707 39954269

[23] Pérez-Castillo ÍM, Williams JA, López-Chicharro J, et al. Compositional aspects of beverages designed to promote hydration before, during, and after exercise: concepts revisited. Nutrients. 2023;16(1):17. doi: 10.3390/nu16010017 . 38201848 PMC10781183

[24] McCubbin AJ, Irwin CG, Costa RJS. Nourishing physical productivity and performance on a warming planet - challenges and nutritional strategies to mitigate exertional heat stress. Curr Nutr Rep. 2024;13(3):399–411. doi: 10.1007/s13668-024-00554-8 38995600 PMC11327203

[25] Wijering LAJ, Cotter JD, Rehrer NJ. A randomized, cross-over trial assessing effects of beverage sodium concentration on plasma sodium concentration and plasma volume during prolonged exercise in the heat. Eur J Appl Physiol. 2023;123(1):81–89. doi: 10.1007/s00421-022-05025-y 36173481 PMC9813217

[26] Li H, Early KS, Zhang G, et al. Personalized hydration strategy to improve fluid balance and intermittent exercise performance in the heat. Nutrients. 2024;16(9):1341. Published 2024 Apr 29. doi: 10.3390/nu16091341 38732589 PMC11085813

[27] Tabuchi S, Horie S, Kawanami S, et al. Efficacy of ice slurry and carbohydrate-electrolyte solutions for firefighters. J Occup Health. 2021;63(1):e12263. doi: 10.1002/1348-9585.12263 34375489 PMC8354579

[28] Ly NQ, Hamstra-Wright KL, Horswill CA. Post-exercise rehydration in athletes: effects of sodium and carbohydrate in commercial hydration beverages. Nutrients. 2023;15(22):4759. Published 2023 Nov 12. doi: 10.3390/nu15224759 38004153 PMC10674530

[29] Peden DL, Funnell MP, Reynolds KM, et al. Post-exercise rehydration: comparing the efficacy of three commercial oral rehydration solutions. Front Sports Act Living. 2023;5:1158167. doi: 10.3389/fspor.2023.1158167 37181252 PMC10174327

[30] García-Heras F, Gutiérrez-Arroyo J, Rodríguez-Medina J, et al. Determinants of health and performance in wildland firefighters: a narrative review. J Funct Morphol Kinesiol. 2025;10(1):80. doi: 10.3390/jfmk10010080 40137332 PMC11943278

[31] Podlogar T, Bolčič T, Cirnski S, et al. Commercially available carbohydrate drink with menthol fails to improve thermal perception or cycling exercise capacity in males. Eur J Sport Sci. 2022;22(11):1705–1713. doi: 10.1080/17461391.2021.1986140 34559601

[32] Tsutsumi Y, Momma H, Ebihara S, et al. L-menthol administration facilitates breathing comfort during exhaustive endurance running and improves running capacity in well-trained runners: a randomized crossover study. Eur J Sport Sci. 2023;23(9):1913–1921. doi: 10.1080/17461391.2022.2115404 35997234

[33] Baillot M, Hue O, Tran TT, et al. Neuromuscular activity during cycling performance in Hot/Dry and Hot/Humid conditions. Life (Basel). 2021;11(11):1149. doi: 10.3390/life11111149 34833025 PMC8623245

[34] Flood TR, Waldron M, Jeffries O. Oral L-menthol reduces thermal sensation, increases work-rate and extends time to exhaustion, in the heat at a fixed rating of perceived exertion. Eur J Appl Physiol. 2017;117(7):1501–1512. doi: 10.1007/s00421-017-3645-6 28508114

[35] Crosby S, Butcher A, McDonald K, et al. Menthol mouth rinsing maintains relative power production during three-minute maximal cycling performance in the heat compared to cold water and placebo rinsing. Int J Environ Res Public Health. 2022;19(6):3527. doi: 10.3390/ijerph19063527 35329209 PMC8949398

[36] Jeffries O, Jibi G, Clark J, et al. Determination of the optimal dose and dosing strategy for effective L-menthol oral rinsing during exercise in hot environments. Eur J Appl Physiol. 2025;125(3):629–638. doi: 10.1007/s00421-024-05609-w 39367885 PMC11889024

[37] Xu L, Han Y, Chen X, et al. Molecular mechanisms underlying menthol binding and activation of TRPM8 ion channel. Nat Commun. 2020;11(1):3790. doi: 10.1038/s41467-020-17582-x 32728032 PMC7391767

[38] Barwood MJ, Gibson OR, Gillis DJ, et al. Menthol as an ergogenic aid for the Tokyo 2021 olympic games: an expert-led consensus statement using the modified delphi method. Sports Med. 2020;50(10):1709–1727. doi: 10.1007/s40279-020-01313-9 32623642 PMC7497433

