# Supplementary material for: Effects of carbohydrate-electrolyte solutions with and without L-menthol on hydration and performance recovery following simulated firefighting exercise
Source: J Int Soc Sports Nutr. 2026 May 19;23(1):2676193. doi: 10.1080/15502783.2026.2676193 (PMC13188569; doi:10.1080/15502783.2026.2676193)

**SUPPLEMENTARY TABLE**

**Table S1.** Participant characteristics.

| **Variable** | **W** | **CES** | **MCES** |
| --- | --- | --- | --- |
| **Age (y)** | 32.8 ± 5.8 | 33.0 ± 4.9 | 32.6 ± 5.9 |
| **Height (cm)** | 175.4 ± 4.2 | 174.1 ± 5.4 | 176.6 ± 3.5 |
| **Body mass (kg)** | 81.7 ± 9.5 | 80.9 ± 7.7 | 81.5 ± 8.5 |
| **BMI** **(kg/m^2^)** | 26.54 ± 2.93 | 26.63 ± 1.70 | 26.15 ± 2.76 |
| **Fat mass (kg)** | 14.6 ± 5.2 | 14.7 ± 3.1 | 15.4 ± 4.3 |
| **Body fat (%)** | 17.7 ± 5.2 | 18.1 ± 2.4 | 18.8 ± 5.1 |
| **Muscle mass (kg)** | 37.1 ± 1.7 | 37.7 ± 2.7 | 37.0 ± 2.9 |
| **Muscle mass (%)** | 45.9 ± 4.7 | 46.7 ± 2.0 | 45.6 ± 3.3 |

Data are presented as mean ± SD (n = 8 per group). Data are presented as mean ± SD. Abbreviations: W, water group; CES, carbohydrate-electrolyte solution group; MCES, menthol-supplemented carbohydrate-electrolyte solution group.

**SUPPLEMENTARY FIGURE**

**Figure S1. Baseline physical performance immediately after the simulated firefighting task, including (A) task completion time and (B) peak heart rate, across the W, CES, and MCES groups prior to rehydration.** Data are presented as mean ± SD (n = 8 per group). W, water group; CES, carbohydrate-electrolyte solution group; MCES, menthol-supplemented carbohydrate-electrolyte solution group.

**Figure S2. Perceived exertion (rating of perceived exertion, RPE) immediately after the simulated firefighting task (T0) in the W, CES, and MCES groups.** Data are presented as mean ± SD (n = 8 per group). W, water group; CES, carbohydrate-electrolyte solution group; MCES, menthol-supplemented carbohydrate-electrolyte solution group; RPE, rating of perceived exertion.

**Figure S1.**

**
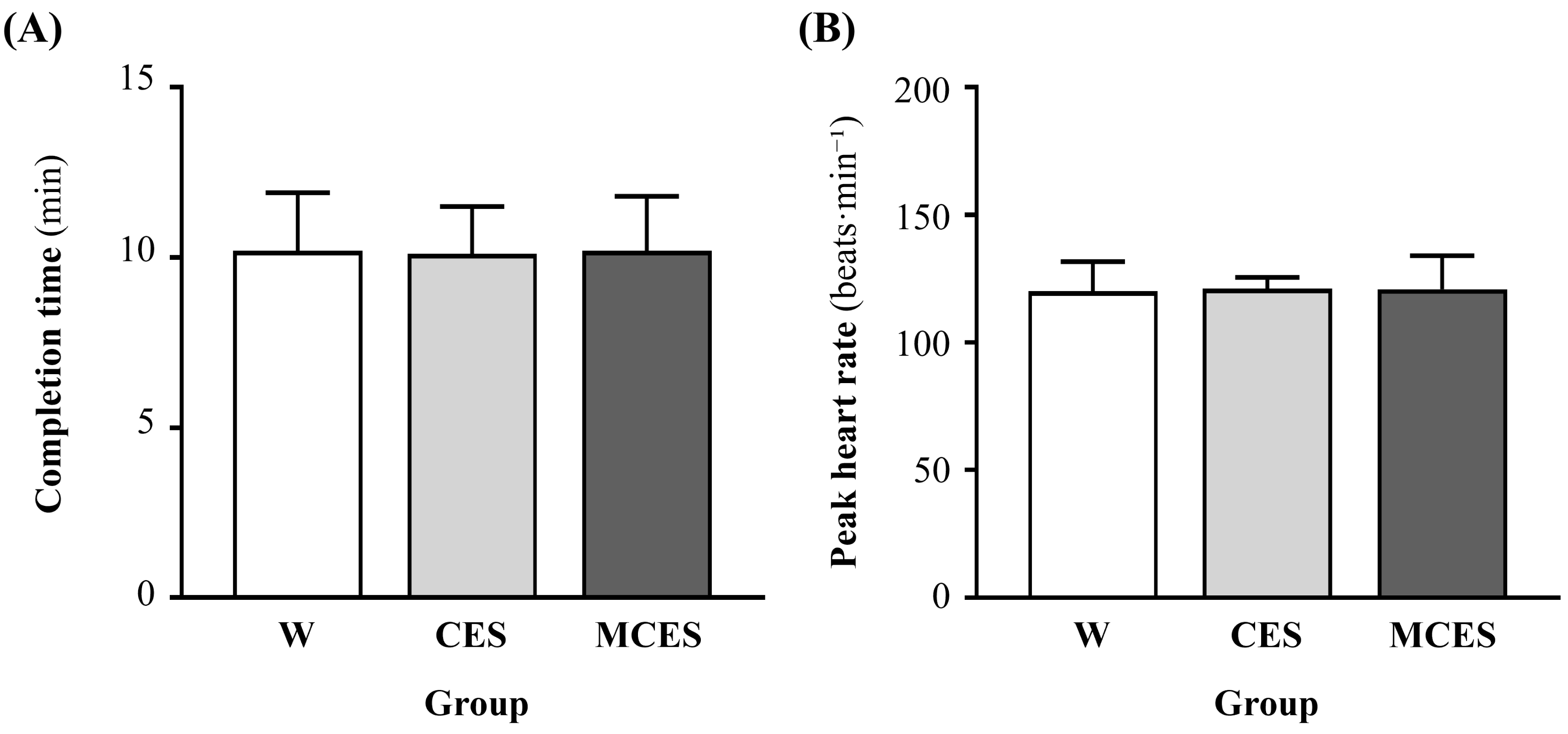
**

**Figure S2**


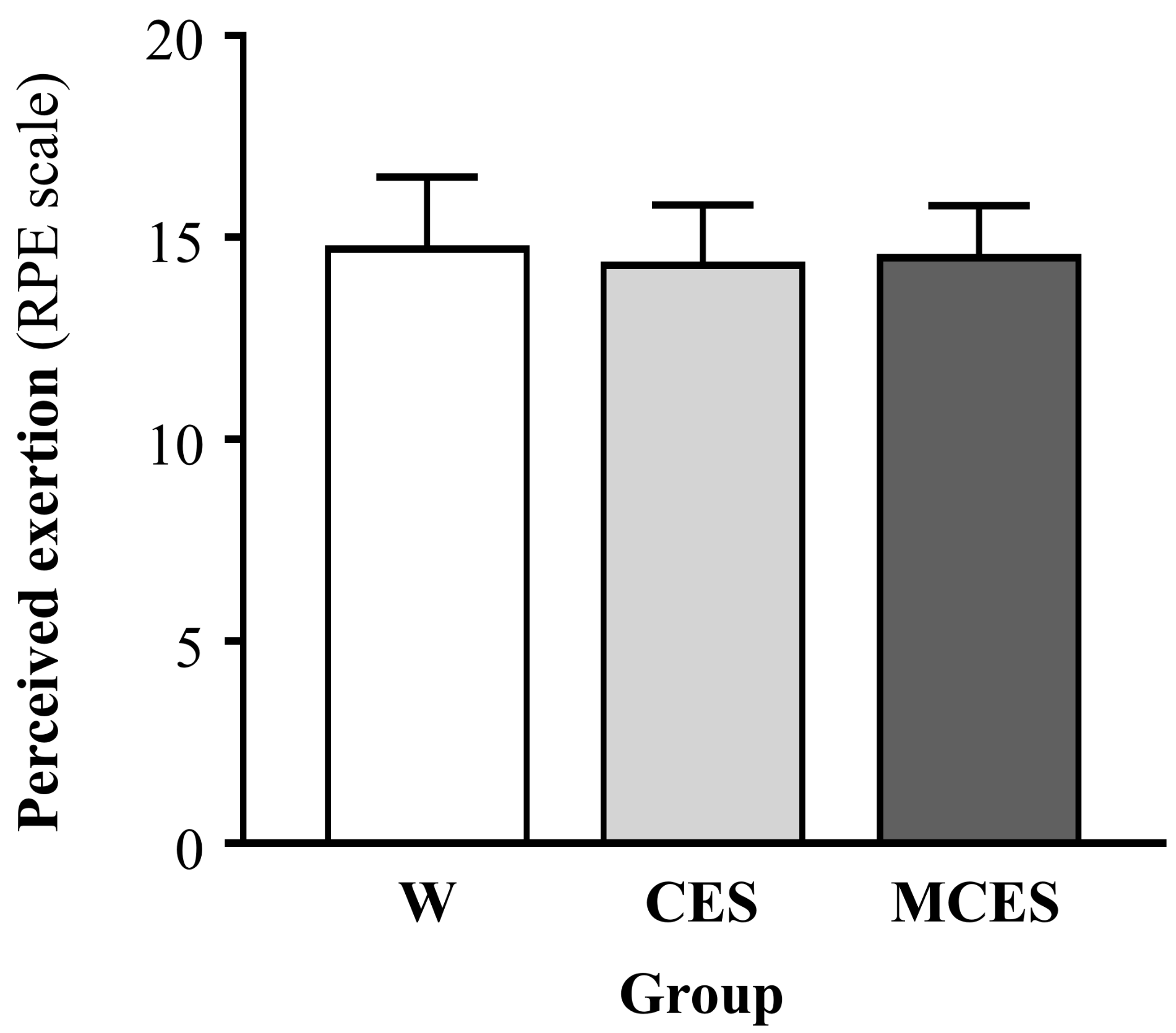

Supplement: Supplementary Material — SUPPLEMENTARY FIGURE TABLE [file RSSN_A_2676193_SM8742.docx]
